# Backboard time for patients receiving spinal immobilization by emergency medical services

**DOI:** 10.1186/1865-1380-6-17

**Published:** 2013-06-20

**Authors:** Derek R Cooney, Harry Wallus, Michael Asaly, Susan Wojcik

**Affiliations:** 1 Department of Emergency Medicine, SUNY Upstate Medical University, 550 East Genesee / EMSTAT Center, Syracuse, NY, 13202, USA; 2 SUNY Upstate Medical University, 750 East Adams Street, Syracuse, NY, 13210, USA

**Keywords:** Backboard, Delay, EMS, ESI score, Immobilization, NEDOCS, Prehospital, Spinal, Spine board, Triage

## Abstract

**Background:**

Use of backboards as part of routine trauma care has recently come into question because of the lack of data to support their effectiveness. Multiple authors have noted the potential harm associated with backboard use, including iatrogenic pain, skin ulceration, increased use of radiographic studies, aspiration and respiratory compromise. An observational study was performed at a level 1 academic trauma center to determine the total and interval backboard times for patients arriving via emergency medical services (EMS).

**Findings:**

Patients were directly observed. Transport time was recorded as an estimate of initiation of backboard use; arrival time, nurse report time and time of removal from the backboard were all recorded. National Emergency Department Overcrowding Study (NEDOCS) score, Emergency Severity Index (ESI) and demographic information were recorded for each patient encounter. Forty-six patients were followed. The mean total backboard time was 54 min (SD ±65). The mean EMS interval was 33 min (SD ±64), and the mean ED interval was 21 min (SD ±15). The ED backboard interval trended inversely to ESI (1 = 5 min, 2 = 10 min, 3 = 25 min, 4 = 26 min, 5 = 32 min).

**Conclusion:**

Patients had a mean total backboard time of around an hour. The mean EMS interval was greater than the mean ED interval. Further study with a larger sample directed to establishing associated factors and to target possible reduction strategies is warranted.

## Background

Use of long spine boards, also known as backboards, for spinal immobilization as part of routine trauma care has recently come under increased scrutiny, and early removal from the board is considered best practice [1]. In addition, multiple authors have brought the utility of backboard use into question because of a lack of data to support their effectiveness in preventing secondary injury and the potential harm associated with backboard use, including iatrogenic pain, skin ulceration, increased use of radiographs, aspiration and respiratory compromise [2-7]. These potential risks prompted initiation of a pilot quality assurance observational study to determine the total and interval backboard times of patients arriving via emergency medical services (EMS) to this level 1 academic trauma center.

## Findings

### Methods

A convenience sample of patients arriving via EMS in spinal immobilization, utilizing a backboard, was included in the study. Trained research associates directly observed patients as they arrived and followed them until they were removed from the backboard. Transport time from EMS documentation was recorded as an estimate of initiation of backboard use. Times of directly observed events, including arrival time, nurse report time and time of removal of the patient from the backboard, were all recorded. At the time of arrival to the ED, research associates also recorded the current National Emergency Department Overcrowding Study (NEDOCS) score for each patient. The Emergency Severity Index (ESI), determined by the nurse, was also added to the data set. Demographic information, including age, race and sex, was also recorded. Data were entered into SPSS® Statistics 19 (IBM®) and analyzed to determine the mean total backboard time, as well as intervals for backboard time associated with EMS transport and the time spent on the backboard in the ED all reported in minutes (min). Data were also analyzed to evaluate for the presence of an association between the NEDOCS score and backboard times. The variables of age and ESI level were also analyzed for associations with backboard times. This manuscript reports the results of quality assurance investigation and was reviewed by the IRB Chief Compliance Officer in reference to the OHRP guidelines. No reference number was assigned as this was considered QA.

### Results

Forty-six patients were followed and times recorded as part of the convenience sample. All data for each patient were complete prior to analysis with SPSS® Statistics 19 (IBM®). The mean total backboard time was 54 min (SD ±65) with a minimum of 11 min and a maximum of 7 h 49 min (in a patient who stayed on the backboard at an outside hospital and was transferred). The mean EMS backboard time interval was 33 min (SD ±64), and the mean ED backboard time interval was 21 min (SD ±15) as summarized in Table 1. Patients varied in age from 9 to greater than 89 years old and were 37% female and 63% male. NEDOCS scores at the time of patient arrival ranged from 36 (busy) to 200 (dangerously overcrowded). The ESI level of patients was predominantly 3 (69.6%), with only two each at levels 4–5 (less emergent) and five each at levels 1–2 (very emergent). There was no statistically significant difference in total time when compared by NEDOCS score grouping (0–100, 101–140, 141–180, ≥181), age group (pediatric = 0 to17 years old and adult = 18 to ≥89 years old) and ESI level. However, the ED backboard interval did show mean increases in time with decreasing severity of triage level by ESI (1 = 5 min, 2 = 10 min, 3 = 25 min, 4 = 26 min, 5 = 32 min) with a statistically significant difference between patients triaged as ESI level 1 and 3 (*p* = 0.035).

**Table 1:** Backboard time with breakdown

	**Mean**	**SD**
**Total**	54 min	±65
**EMS interval**	33 min	±64
**Hospital interval**	21 min	±15

### Study limitations

This represents a small pilot observational quality assurance study. The small sample size in this study limits data analysis. A larger sample size study may reveal associations with the NEDOCS score and ESI, as well as other factors relating to delays in removing patients from backboards in the ED.

### Discussion

In light of the potential harm caused by the use of backboards for immobilization, every effort should be made to ensure that the time is minimized [1]. A study on backboard use by Lerner et al. showed that the mean total ED backboard time was as high as 165.3 min (SD ±49.7) [8]. Other studies have shown successful use of prehosptial algorithms designed to allow EMS providers to avoid spinal immobilization in patients with little to no risk of spinal injury [9-13]. More study in this area is needed to determine causes of delay in removal of patients from the backboard after EMS arrival to the hospital.

## Conclusion

Patients presenting via EMS to this level 1 academic trauma center had a mean total backboard time of nearly an hour. Although the mean EMS interval was greater than the mean ED interval, the ED interval was still significant, with a mean of greater than 20 min. Patients perceived to be in lesser need of emergency care may have had a longer wait until removal from the backboard than those with an obvious need for immediate attention. Further study is needed to elucidate factors associated with delays and to evaluate strategies to reduce total backboard time.

## Abbreviations

ED: Emergency department; EMS: Emergency medicinal services; ESI: Emergency severity index; NEDOCS: National emergency department overcrowding study.

## Competing interests

The authors declare that they have no competing interests. No funds were received in the support of this study.

## Authors’ contributions

DRC designed the study and, along with HW, reviewed the results and prepared the manuscript. MA assisted in data collection and data entry. SW provided design support, statistical analysis and editorial support services to the project. All authors read and approved the final manuscript.

## Authors’ information

DRC is an Associate Professor of Emergency Medicine and the Program Director for the EMS Medicine Fellowship. HW is a Clinical Instructor of Emergency Medicine and was a Fellow in EMS Medicine at the time of study completion. MA was a medical student at the time of the study. SW is an Associate Professor (PhD) and a member of the research faculty.
